# 2-Amino-6-{[(6-chloropyridin-3-yl)methyl](ethyl)amino}-1-methyl-5-nitro-4-phenyl-1,4-dihydro­pyridine-3-carbonitrile ethanol monosolvate

**DOI:** 10.1107/S1600536812011750

**Published:** 2012-03-24

**Authors:** Chuan-Wen Sun, Yan-Xia Chen, Tian-Yan Liu

**Affiliations:** a Department of Chemistry, College of Life and Environmental Science, Shanghai Normal University, Shanghai 200234, People’s Republic of China

## Abstract

In the title compound, C_21_H_21_ClN_6_O_2_·C_2_H_6_O, a member of the insecticidal active neonicotinoid group of compounds, the 1,4-dihydro­pyridine ring adopts a boat conformation. An intra­molecular C—H⋯O hydrogen bond occurs while the components are linked by an N—H⋯O interaction. The crystal packing is stablized by O—H⋯N hydrogen bonds and C—H⋯O interactions.

## Related literature

For the synthesis, see: Zhang *et al.* (2010 ▶). For the insectidal activity of nitenpyram [systematic name: (*E*)-*N*-(6-Chloro-3-pyridyl­meth­yl)-*N*-ethyl-*N*′-methyl-2-nitro­vinyl­idenediamine], see: Elbert & Nauen (2000 ▶); Jeschke & Nauen (2008 ▶); Kashiwada (1996 ▶); Minamida *et al.* (1993 ▶); Shao *et al.* (2008 ▶); Tomizawa & Casida (2009 ▶).
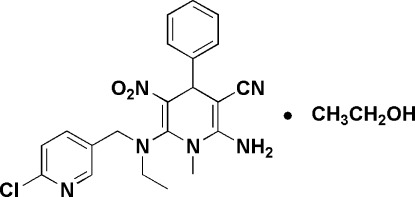


## Experimental

### 

#### Crystal data

C_21_H_21_ClN_6_O_2_·C_2_H_6_O
*M* *_r_* = 470.96
Orthorhombic, 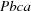

*a* = 19.3334 (19) Å
*b* = 12.1156 (12) Å
*c* = 20.644 (2) Å
*V* = 4835.5 (8) Å^3^
*Z* = 8
Mo *K*α radiation
μ = 0.19 mm^−1^
*T* = 298 K
0.16 × 0.12 × 0.10 mm

#### Data collection

Bruker SMART CCD area-detector diffractometer
Absorption correction: multi-scan (*SADABS*: Bruker, 2001 ▶) *T*_min_ = 0.970, *T*_max_ = 0.981
44471 measured reflections
4267 independent reflections
3044 reflections with *I* > 2σ(*I*)
*R*_int_ = 0.045

#### Refinement

*R*[*F* ^2^ > 2σ(*F* ^2^)] = 0.048
*wR*(*F* ^2^) = 0.135
*S* = 1.05
4267 reflections
330 parameters
4 restraints
H-atom parameters constrained
Δρ_max_ = 0.21 e Å^−3^
Δρ_min_ = −0.20 e Å^−3^

### 

Data collection: *SMART* (Bruker, 2001 ▶); cell refinement: *SAINT-Plus* (Bruker, 2001 ▶); data reduction: *SAINT-Plus*; program(s) used to solve structure: *SHELXS97* (Sheldrick, 2008 ▶); program(s) used to refine structure: *SHELXL97* (Sheldrick, 2008 ▶); molecular graphics: *PLATON* (Spek, 2009 ▶); software used to prepare material for publication: *PLATON*.

## Supplementary Material

Crystal structure: contains datablock(s) I, global. DOI: 10.1107/S1600536812011750/gg2077sup1.cif

Structure factors: contains datablock(s) I. DOI: 10.1107/S1600536812011750/gg2077Isup2.hkl

Supplementary material file. DOI: 10.1107/S1600536812011750/gg2077Isup3.cml

Additional supplementary materials:  crystallographic information; 3D view; checkCIF report

## Figures and Tables

**Table 1:** Hydrogen-bond geometry (Å, °)

*D*—H⋯*A*	*D*—H	H⋯*A*	*D*⋯*A*	*D*—H⋯*A*
O3—H3*A*⋯N1^i^	0.82	1.97	2.785 (13)	179
N4—H4*B*⋯O3	0.86	2.26	2.902 (11)	132
C6—H6*A*⋯O2	0.97	2.13	2.783 (3)	124
C7—H7*B*⋯O2^ii^	0.97	2.57	3.378 (3)	141

Symmetry codes: (i) 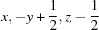; (ii) 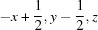.

**Fig. 1.**
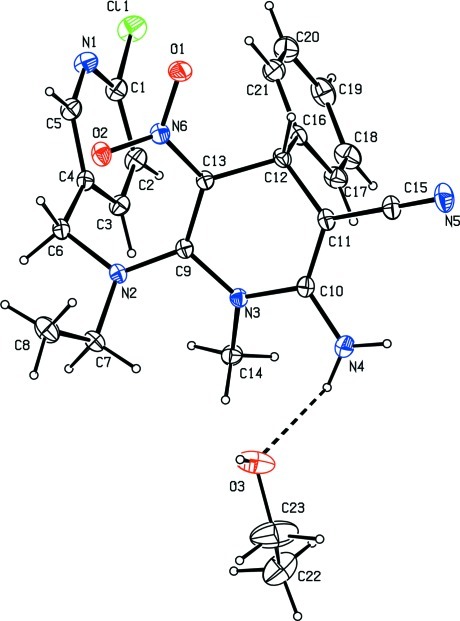
The molecular structure of (I) shown with 30% displacement ellipsoids. For clarity, the minor component of the disordered ethanol molecule is omitted. Hydrogen bonding is shown as dashed lines.

**Fig. 2.**
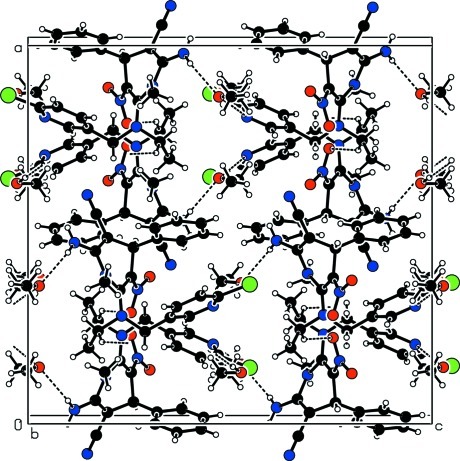
Part of the crystal packing in the title compound (I). Hydrogen bonds are shown as dashed lines.

## supplementary crystallographic information

### Comment

Neonicotinoid insecticides (NNSs), which act agonistically on the insect nicotinic acetylcholine receptors (nAChRs), are gaining widespread use as a way to control pests, because of their high potency and low mammalian toxicity. As part of the chloronicotinyl subclass, nitenpyram, which was brought to the market two decades ago, also showed higher selectivity and better systemic properties against mammals, birds, aquatic life than insects, due to the differential binding affinities with the nAChR receptors of their neurosystem. (Jeschke & Nauen, 2008; Tomizawa & Casida, 2009; Minamida *et al.*, 1993; Kashiwada, 1996; Shao *et al.*, 2008; Elbert & Nauen, 2000). In this report, the title compound (Scheme I) was synthesized and characterized by X-ray diffraction.

In the title structure, C_21_H_21_ClN_6_O_2_C_2_H_6_O, (I), there is a cis-2-Amino-6-[N-(6-chloro-3-pyridinylmethyl)-N-ethyl]amino-3-cyano-1-methyl-5- nitro-4-phenyl-1,4-dihydropyridine molecule and a ethanol molecule in the asymmetric unit (Fig. 1). The 1,4-dihydropyridine ring adopts a sofa (boat) conformation. As compared with the trans configuration of nitro in the crystal structure of nitenpyram, the nitro group in the title compound is in the cis configuration as anticipated. Interestingly, the C–C and C-N bond length data (C9–N3 1.389 (2) Å, N3–C10 1.402 (3) Å, C11–C12 1.510 (3) Å and C12-C13 1.519 (3) Å) in the structure of (I) are shorter than the standard C–C (1.54 Å) and C–N (1.47 Å). On the contrary, the C═C bond length data (C9═C13 1.384 (3) Å and C10═C11 1.348 (3) Å) are longer than the standard C═C bond (1.34 Å). This shows that there is a homo-conjugation effect on the 1,4-dihydropyridine scaffold (Fig. 1).

The crystal packing is stablized by O-H···N, N-H···O and C-H···O hydrogen bonds (Fig. 2). Analysis shows that no intermolecular p···π or C-H···π interactions exist in the crystal structure.

### Experimental

The title compound was prepared by the literature method (Zhang *et al.*, 2010) and it was obtained using volatilization of petroleum ether and ethanol solution at room temperature, giving yellow crystals (yield 83.7%). ^1^H NMR (CDCl_3_, 400 Hz): 8.08 (d, *J* = 12.4 Hz,1*H*, Py—H), 7.34 (d, *J* = 8.9 Hz, 1*H*, Py—H), 7.24 (s,3*H*,Ph—H), 7.08 (m, *J* = 7.8 Hz, 1*H*, Py—H), 7.05–6.95 (m, 2*H*, Ph—H), 5.06 (s, 1*H*, CH), 4.79 (s, 2*H*, NH_2_), 4.33 (d, *J* = 14.8 Hz, 1*H*), 4.06 (m, *J* = 14.6 Hz, 1*H*), 3.35–3.20 (m, 1*H*), 3.17 (s, 3*H*, NCH_3_), 3.10 (d, *J* = 7.3 Hz,1*H*), 1.33–1.21 (m,3*H*,NCH_2_CH_3_). IR(KBr, cm^-1^) 2974 (CH_3_), 3327, 3197 (NH_2_), 2184 (CN), 1457, 1409 (NO_2_), 1648, 1614, 1557 (benzene).Anal. calcd. for C_23_H_21_ClN_6_O_2_ C 59.36, H 4.98, N 19.78% found, C 59.38, H 4.97, N 19.76%.

### Refinement

During the refinement, the ethanol molecule was disordered over two sites. These C-C and C-O distances were refined with the restraints of C-C = 1.51 (1)Å and C-O = 1.38 (1)Å by using the DFIX command. The final occupancies for the major and minor components were 0.57 (1):0.43 (1), respectively. In (I), H atoms bonded to C and N atoms were located at their ideal positions and subsequently treated as riding modes with C–H distances of 0.93Å (aromatic), 0.97Å (methylene) 0.98Å (methine) 0.86Å (amine) and 0.96Å (methyl) with *U*_iso_(H) = 1.2*U*_eq_(aromatic, methylene, methine C or N) or 1.5*U*_eq_(methyl C). H atoms bonded to ethanol O atoms were located at its ideal position (O-H=0.82Å) and refined with the constraint of the *U*_iso_(H) = 1.5*U*_eq_(O).

### Crystal data

**Table:**

C_21_H_21_ClN_6_O_2_·C_2_H_6_O	*F*(000) = 1984
*M**_r_* = 470.96	*D*_x_ = 1.294 Mg m^−^^3^
Orthorhombic, *P**b**c**a*	Mo *K*α radiation, λ = 0.71073 Å
Hall symbol: -P 2ac 2ab	Cell parameters from 5913 reflections
*a* = 19.3334 (19) Å	θ = 2.2–20.1°
*b* = 12.1156 (12) Å	µ = 0.19 mm^−^^1^
*c* = 20.644 (2) Å	*T* = 298 K
*V* = 4835.5 (8) Å^3^	Block, yellow
*Z* = 8	0.16 × 0.12 × 0.10 mm

### Data collection

**Table:**

Bruker SMART CCD area-detector diffractometer	4267 independent reflections
Radiation source: fine-focus sealed tube	3044 reflections with *I* > 2σ(*I*)
Graphite monochromator	*R*_int_ = 0.045
phi and ω scans	θ_max_ = 25.0°, θ_min_ = 2.0°
Absorption correction: multi-scan (*SADABS*: Bruker, 2001)	*h* = −23→23
*T*_min_ = 0.970, *T*_max_ = 0.981	*k* = −14→14
44471 measured reflections	*l* = −24→24

### Refinement

**Table:**

Refinement on *F*^2^	Primary atom site location: structure-invariant direct methods
Least-squares matrix: full	Secondary atom site location: difference Fourier map
*R*[*F*^2^ > 2σ(*F*^2^)] = 0.048	Hydrogen site location: inferred from neighbouring sites
*wR*(*F*^2^) = 0.135	H-atom parameters constrained
*S* = 1.05	*w* = 1/[σ^2^(*F*_o_^2^) + (0.0687*P*)^2^ + 0.8771*P*] where *P* = (*F*_o_^2^ + 2*F*_c_^2^)/3
4267 reflections	(Δ/σ)_max_ < 0.001
330 parameters	Δρ_max_ = 0.21 e Å^−^^3^
4 restraints	Δρ_min_ = −0.20 e Å^−^^3^

### Special details

**Table:**

Geometry. All esds (except the esd in the dihedral angle between two l.s. planes) are estimated using the full covariance matrix. The cell esds are taken into account individually in the estimation of esds in distances, angles and torsion angles; correlations between esds in cell parameters are only used when they are defined by crystal symmetry. An approximate (isotropic) treatment of cell esds is used for estimating esds involving l.s. planes.
Refinement. Refinement of F^2^ against ALL reflections. The weighted R-factor wR and goodness of fit S are based on F^2^, conventional R-factors R are based on F, with F set to zero for negative F^2^. The threshold expression of F^2^ > 2sigma(F^2^) is used only for calculating R-factors(gt) etc. and is not relevant to the choice of reflections for refinement. R-factors based on F^2^ are statistically about twice as large as those based on F, and R- factors based on ALL data will be even larger.

### Fractional atomic coordinates and isotropic or equivalent isotropic displacement parameters (Å^2^)

**Table:**

	*x*	*y*	*z*	*U*_iso_*/*U*_eq_	Occ. (<1)
C1	0.16697 (13)	0.1575 (2)	0.97037 (12)	0.0672 (6)	
C2	0.16346 (13)	0.0715 (2)	0.92705 (12)	0.0677 (6)	
H2	0.1423	0.0051	0.9379	0.081*	
C3	0.19239 (11)	0.08776 (18)	0.86736 (11)	0.0594 (6)	
H3	0.1913	0.0314	0.8368	0.071*	
C4	0.22332 (10)	0.18758 (16)	0.85211 (10)	0.0509 (5)	
C5	0.22151 (11)	0.26760 (19)	0.89890 (12)	0.0636 (6)	
H5	0.2408	0.3359	0.8890	0.076*	
C6	0.26080 (10)	0.20673 (18)	0.78962 (10)	0.0538 (5)	
H6A	0.2766	0.2827	0.7884	0.065*	
H6B	0.3014	0.1596	0.7884	0.065*	
C7	0.25784 (11)	0.15403 (18)	0.67342 (11)	0.0608 (6)	
H7A	0.2256	0.1348	0.6392	0.073*	
H7B	0.2857	0.0893	0.6828	0.073*	
C8	0.30456 (13)	0.2463 (3)	0.65010 (14)	0.0890 (8)	
H8A	0.2773	0.3106	0.6409	0.133*	
H8B	0.3282	0.2233	0.6115	0.133*	
H8C	0.3379	0.2634	0.6831	0.133*	
C9	0.15149 (10)	0.21488 (15)	0.72709 (10)	0.0457 (5)	
C10	0.04705 (11)	0.17912 (18)	0.66673 (10)	0.0540 (5)	
C11	0.01366 (10)	0.26259 (18)	0.69650 (11)	0.0555 (5)	
C12	0.04016 (10)	0.30390 (16)	0.76091 (10)	0.0515 (5)	
H12	0.0249	0.3805	0.7660	0.062*	
C13	0.11850 (9)	0.30414 (15)	0.75527 (10)	0.0473 (5)	
C14	0.12560 (12)	0.02347 (17)	0.68983 (12)	0.0629 (6)	
H14A	0.1431	0.0043	0.6478	0.094*	
H14B	0.0841	−0.0175	0.6983	0.094*	
H14C	0.1596	0.0060	0.7221	0.094*	
C15	−0.04783 (12)	0.3063 (2)	0.66890 (13)	0.0663 (6)	
C16	0.01488 (10)	0.23895 (18)	0.81946 (11)	0.0551 (5)	
C17	−0.01453 (13)	0.1358 (2)	0.81419 (14)	0.0727 (7)	
H17	−0.0206	0.1046	0.7734	0.087*	
C18	−0.03510 (15)	0.0781 (2)	0.86844 (16)	0.0910 (9)	
H18	−0.0544	0.0082	0.8638	0.109*	
C19	−0.02752 (15)	0.1221 (3)	0.92841 (17)	0.0951 (9)	
H19	−0.0416	0.0830	0.9649	0.114*	
C20	0.00120 (15)	0.2249 (3)	0.93454 (15)	0.0975 (10)	
H20	0.0067	0.2558	0.9755	0.117*	
C21	0.02208 (13)	0.2832 (2)	0.88048 (13)	0.0784 (7)	
H21	0.0412	0.3531	0.8854	0.094*	
Cl1	0.13433 (5)	0.14060 (7)	1.04868 (3)	0.1000 (3)	
N1	0.19380 (11)	0.25445 (17)	0.95814 (10)	0.0710 (6)	
N2	0.21881 (8)	0.18511 (13)	0.73179 (8)	0.0486 (4)	
N3	0.11034 (8)	0.14244 (13)	0.69193 (8)	0.0491 (4)	
N4	0.02386 (10)	0.12143 (17)	0.61498 (10)	0.0750 (6)	
H4A	−0.0156	0.1370	0.5981	0.090*	
H4B	0.0486	0.0692	0.5989	0.090*	
N5	−0.09771 (11)	0.34154 (19)	0.64678 (13)	0.0927 (7)	
N6	0.15343 (9)	0.40156 (14)	0.77108 (9)	0.0568 (5)	
O1	0.12139 (9)	0.47446 (13)	0.80126 (9)	0.0812 (5)	
O2	0.21474 (8)	0.41614 (12)	0.75479 (9)	0.0703 (5)	
C22	0.1322 (6)	−0.1213 (7)	0.4967 (5)	0.147 (4)	0.57
H22A	0.1746	−0.1413	0.5178	0.220*	0.57
H22B	0.1263	−0.1656	0.4585	0.220*	0.57
H22C	0.0941	−0.1334	0.5256	0.220*	0.57
C23	0.1345 (8)	−0.0106 (7)	0.4793 (5)	0.194 (6)	0.57
H23A	0.0942	0.0043	0.4528	0.233*	0.57
H23B	0.1747	−0.0004	0.4519	0.233*	0.57
O3	0.1370 (7)	0.0705 (9)	0.5276 (6)	0.145 (5)	0.57
H3A	0.1532	0.1222	0.5069	0.218*	0.57
C22'	0.1377 (5)	−0.0766 (13)	0.4602 (5)	0.113 (3)	0.43
H22D	0.0896	−0.0859	0.4701	0.169*	0.43
H22E	0.1581	−0.1473	0.4513	0.169*	0.43
H22F	0.1425	−0.0298	0.4229	0.169*	0.43
C23'	0.1723 (4)	−0.0269 (7)	0.5146 (4)	0.094 (2)	0.43
H23C	0.1732	−0.0788	0.5503	0.113*	0.43
H23D	0.2198	−0.0105	0.5028	0.113*	0.43
O3'	0.1403 (5)	0.0683 (7)	0.5341 (6)	0.085 (3)	0.43
H3B	0.1193	0.0955	0.5035	0.127*	0.43

### Atomic displacement parameters (Å^2^)

**Table:**

	*U*^11^	*U*^22^	*U*^33^	*U*^12^	*U*^13^	*U*^23^
C1	0.0703 (15)	0.0707 (16)	0.0606 (14)	−0.0110 (12)	0.0025 (12)	0.0082 (12)
C2	0.0759 (15)	0.0581 (14)	0.0690 (16)	−0.0160 (12)	−0.0023 (13)	0.0120 (12)
C3	0.0652 (14)	0.0515 (12)	0.0616 (14)	−0.0049 (10)	−0.0046 (11)	0.0034 (11)
C4	0.0448 (11)	0.0496 (12)	0.0582 (13)	−0.0018 (9)	−0.0067 (9)	0.0043 (10)
C5	0.0669 (14)	0.0556 (13)	0.0683 (16)	−0.0134 (11)	0.0021 (12)	0.0053 (12)
C6	0.0416 (11)	0.0535 (12)	0.0665 (14)	−0.0009 (9)	−0.0016 (10)	0.0037 (10)
C7	0.0543 (12)	0.0631 (14)	0.0651 (14)	0.0088 (10)	0.0134 (11)	0.0066 (11)
C8	0.0765 (17)	0.0963 (19)	0.094 (2)	−0.0013 (15)	0.0331 (15)	0.0180 (16)
C9	0.0440 (11)	0.0428 (11)	0.0505 (11)	−0.0034 (9)	0.0030 (9)	0.0091 (9)
C10	0.0472 (11)	0.0586 (13)	0.0563 (13)	−0.0054 (10)	−0.0024 (10)	0.0052 (11)
C11	0.0430 (11)	0.0554 (12)	0.0682 (14)	0.0019 (10)	−0.0014 (10)	0.0085 (11)
C12	0.0435 (11)	0.0430 (11)	0.0682 (14)	0.0065 (9)	0.0034 (10)	0.0016 (10)
C13	0.0448 (11)	0.0389 (10)	0.0584 (12)	−0.0016 (9)	0.0009 (9)	0.0053 (9)
C14	0.0681 (14)	0.0443 (12)	0.0764 (16)	−0.0008 (10)	−0.0046 (12)	−0.0002 (11)
C15	0.0502 (13)	0.0630 (14)	0.0856 (17)	−0.0020 (11)	−0.0027 (12)	0.0091 (13)
C16	0.0394 (10)	0.0570 (13)	0.0690 (14)	0.0056 (10)	0.0088 (10)	0.0011 (11)
C17	0.0740 (15)	0.0651 (15)	0.0790 (17)	−0.0060 (13)	0.0211 (13)	0.0025 (13)
C18	0.094 (2)	0.0777 (18)	0.102 (2)	−0.0085 (15)	0.0348 (18)	0.0158 (17)
C19	0.0780 (19)	0.118 (3)	0.090 (2)	0.0036 (18)	0.0234 (16)	0.033 (2)
C20	0.087 (2)	0.141 (3)	0.0646 (18)	−0.006 (2)	0.0106 (15)	0.0001 (19)
C21	0.0729 (16)	0.0889 (19)	0.0735 (18)	−0.0101 (14)	0.0092 (14)	−0.0073 (15)
Cl1	0.1254 (7)	0.1083 (6)	0.0663 (5)	−0.0273 (5)	0.0183 (4)	0.0100 (4)
N1	0.0846 (14)	0.0667 (13)	0.0618 (13)	−0.0142 (11)	0.0057 (10)	−0.0010 (10)
N2	0.0411 (9)	0.0498 (9)	0.0550 (10)	0.0011 (7)	0.0036 (8)	0.0046 (8)
N3	0.0455 (9)	0.0430 (9)	0.0588 (10)	0.0013 (7)	−0.0006 (8)	0.0022 (8)
N4	0.0625 (12)	0.0837 (14)	0.0787 (14)	0.0041 (10)	−0.0180 (10)	−0.0155 (12)
N5	0.0568 (13)	0.0909 (16)	0.130 (2)	0.0106 (11)	−0.0179 (13)	0.0186 (15)
N6	0.0557 (11)	0.0431 (10)	0.0715 (12)	−0.0019 (9)	0.0003 (9)	0.0057 (9)
O1	0.0788 (11)	0.0533 (10)	0.1115 (14)	−0.0030 (9)	0.0099 (10)	−0.0224 (10)
O2	0.0534 (9)	0.0533 (9)	0.1042 (13)	−0.0117 (7)	0.0058 (9)	0.0071 (9)
C22	0.203 (10)	0.110 (7)	0.128 (8)	−0.023 (6)	−0.051 (7)	0.017 (5)
C23	0.363 (19)	0.097 (7)	0.122 (8)	0.046 (9)	−0.026 (10)	−0.009 (6)
O3	0.219 (11)	0.122 (8)	0.095 (6)	0.048 (7)	0.036 (6)	−0.014 (6)
C22'	0.099 (6)	0.134 (10)	0.105 (8)	−0.003 (7)	0.013 (6)	−0.021 (7)
C23'	0.099 (5)	0.105 (7)	0.077 (5)	0.040 (5)	−0.010 (4)	0.013 (5)
O3'	0.105 (6)	0.055 (5)	0.093 (7)	−0.001 (5)	0.037 (5)	0.016 (5)

### Geometric parameters (Å, º)

**Table:**

C1—N1	1.309 (3)	C14—H14B	0.9600
C1—C2	1.374 (3)	C14—H14C	0.9600
C1—Cl1	1.747 (3)	C15—N5	1.149 (3)
C2—C3	1.368 (3)	C16—C21	1.376 (3)
C2—H2	0.9300	C16—C17	1.377 (3)
C3—C4	1.385 (3)	C17—C18	1.379 (4)
C3—H3	0.9300	C17—H17	0.9300
C4—C5	1.369 (3)	C18—C19	1.356 (4)
C4—C6	1.498 (3)	C18—H18	0.9300
C5—N1	1.345 (3)	C19—C20	1.370 (5)
C5—H5	0.9300	C19—H19	0.9300
C6—N2	1.467 (3)	C20—C21	1.381 (4)
C6—H6A	0.9700	C20—H20	0.9300
C6—H6B	0.9700	C21—H21	0.9300
C7—N2	1.471 (3)	N4—H4A	0.8600
C7—C8	1.516 (3)	N4—H4B	0.8600
C7—H7A	0.9700	N6—O2	1.245 (2)
C7—H7B	0.9700	N6—O1	1.246 (2)
C8—H8A	0.9600	C22—C23	1.388 (8)
C8—H8B	0.9600	C22—H22A	0.9600
C8—H8C	0.9600	C22—H22B	0.9600
C9—N2	1.354 (2)	C22—H22C	0.9600
C9—C13	1.384 (3)	C23—O3	1.400 (9)
C9—N3	1.389 (2)	C23—H23A	0.9700
C10—C11	1.348 (3)	C23—H23B	0.9700
C10—N4	1.353 (3)	O3—H3A	0.8200
C10—N3	1.402 (3)	C22'—C23'	1.439 (8)
C11—C15	1.420 (3)	C22'—H22D	0.9600
C11—C12	1.510 (3)	C22'—H22E	0.9600
C12—C13	1.519 (3)	C22'—H22F	0.9600
C12—C16	1.523 (3)	C23'—O3'	1.370 (8)
C12—H12	0.9800	C23'—H23C	0.9700
C13—N6	1.398 (3)	C23'—H23D	0.9700
C14—N3	1.472 (3)	O3'—H3A	0.8975
C14—H14A	0.9600	O3'—H3B	0.8200
			
N1—C1—C2	125.0 (2)	H14A—C14—H14C	109.5
N1—C1—Cl1	115.25 (19)	H14B—C14—H14C	109.5
C2—C1—Cl1	119.72 (19)	N5—C15—C11	179.7 (3)
C3—C2—C1	117.2 (2)	C21—C16—C17	117.9 (2)
C3—C2—H2	121.4	C21—C16—C12	119.5 (2)
C1—C2—H2	121.4	C17—C16—C12	122.6 (2)
C2—C3—C4	120.4 (2)	C16—C17—C18	121.0 (3)
C2—C3—H3	119.8	C16—C17—H17	119.5
C4—C3—H3	119.8	C18—C17—H17	119.5
C5—C4—C3	116.5 (2)	C19—C18—C17	120.8 (3)
C5—C4—C6	120.69 (19)	C19—C18—H18	119.6
C3—C4—C6	122.7 (2)	C17—C18—H18	119.6
N1—C5—C4	124.6 (2)	C18—C19—C20	119.0 (3)
N1—C5—H5	117.7	C18—C19—H19	120.5
C4—C5—H5	117.7	C20—C19—H19	120.5
N2—C6—C4	113.92 (16)	C19—C20—C21	120.6 (3)
N2—C6—H6A	108.8	C19—C20—H20	119.7
C4—C6—H6A	108.8	C21—C20—H20	119.7
N2—C6—H6B	108.8	C16—C21—C20	120.7 (3)
C4—C6—H6B	108.8	C16—C21—H21	119.6
H6A—C6—H6B	107.7	C20—C21—H21	119.6
N2—C7—C8	112.2 (2)	C1—N1—C5	116.1 (2)
N2—C7—H7A	109.2	C9—N2—C6	122.86 (17)
C8—C7—H7A	109.2	C9—N2—C7	120.18 (18)
N2—C7—H7B	109.2	C6—N2—C7	115.36 (15)
C8—C7—H7B	109.2	C9—N3—C10	119.57 (16)
H7A—C7—H7B	107.9	C9—N3—C14	121.28 (16)
C7—C8—H8A	109.5	C10—N3—C14	118.32 (17)
C7—C8—H8B	109.5	C10—N4—H4A	120.0
H8A—C8—H8B	109.5	C10—N4—H4B	120.0
C7—C8—H8C	109.5	H4A—N4—H4B	120.0
H8A—C8—H8C	109.5	O2—N6—O1	120.54 (17)
H8B—C8—H8C	109.5	O2—N6—C13	121.09 (18)
N2—C9—C13	128.39 (18)	O1—N6—C13	118.36 (17)
N2—C9—N3	114.80 (17)	C22—C23—O3	119.7 (11)
C13—C9—N3	116.72 (17)	C22—C23—H23A	107.4
C11—C10—N4	126.1 (2)	O3—C23—H23A	107.4
C11—C10—N3	119.12 (19)	C22—C23—H23B	107.4
N4—C10—N3	114.74 (19)	O3—C23—H23B	107.4
C10—C11—C15	119.8 (2)	H23A—C23—H23B	106.9
C10—C11—C12	119.17 (18)	C23—O3—H3A	100.2
C15—C11—C12	120.9 (2)	C23'—C22'—H22D	109.5
C11—C12—C13	105.76 (17)	C23'—C22'—H22E	109.5
C11—C12—C16	114.75 (17)	H22D—C22'—H22E	109.5
C13—C12—C16	112.45 (17)	C23'—C22'—H22F	109.5
C11—C12—H12	107.9	H22D—C22'—H22F	109.5
C13—C12—H12	107.9	H22E—C22'—H22F	109.5
C16—C12—H12	107.9	O3'—C23'—C22'	111.9 (11)
C9—C13—N6	122.37 (17)	O3'—C23'—H23C	109.2
C9—C13—C12	119.35 (17)	C22'—C23'—H23C	109.2
N6—C13—C12	117.72 (17)	O3'—C23'—H23D	109.2
N3—C14—H14A	109.5	C22'—C23'—H23D	109.2
N3—C14—H14B	109.5	H23C—C23'—H23D	107.9
H14A—C14—H14B	109.5	C23'—O3'—H3A	107.5
N3—C14—H14C	109.5	C23'—O3'—H3B	109.5
			
N1—C1—C2—C3	2.3 (4)	C12—C16—C17—C18	178.2 (2)
Cl1—C1—C2—C3	−177.31 (18)	C16—C17—C18—C19	0.7 (4)
C1—C2—C3—C4	−0.4 (3)	C17—C18—C19—C20	−0.2 (5)
C2—C3—C4—C5	−1.5 (3)	C18—C19—C20—C21	0.1 (5)
C2—C3—C4—C6	174.9 (2)	C17—C16—C21—C20	0.8 (4)
C3—C4—C5—N1	1.9 (3)	C12—C16—C21—C20	−178.4 (2)
C6—C4—C5—N1	−174.6 (2)	C19—C20—C21—C16	−0.4 (4)
C5—C4—C6—N2	−127.6 (2)	C2—C1—N1—C5	−2.1 (4)
C3—C4—C6—N2	56.2 (3)	Cl1—C1—N1—C5	177.59 (17)
N4—C10—C11—C15	−8.0 (3)	C4—C5—N1—C1	−0.1 (4)
N3—C10—C11—C15	175.28 (19)	C13—C9—N2—C6	31.1 (3)
N4—C10—C11—C12	168.5 (2)	N3—C9—N2—C6	−145.33 (18)
N3—C10—C11—C12	−8.2 (3)	C13—C9—N2—C7	−133.8 (2)
C10—C11—C12—C13	40.0 (2)	N3—C9—N2—C7	49.8 (2)
C15—C11—C12—C13	−143.46 (19)	C4—C6—N2—C9	40.7 (3)
C10—C11—C12—C16	−84.5 (2)	C4—C6—N2—C7	−153.71 (17)
C15—C11—C12—C16	92.0 (2)	C8—C7—N2—C9	101.8 (2)
N2—C9—C13—N6	27.3 (3)	C8—C7—N2—C6	−64.2 (2)
N3—C9—C13—N6	−156.32 (18)	N2—C9—N3—C10	−160.22 (17)
N2—C9—C13—C12	−161.53 (19)	C13—C9—N3—C10	22.9 (3)
N3—C9—C13—C12	14.9 (3)	N2—C9—N3—C14	30.4 (3)
C11—C12—C13—C9	−43.8 (2)	C13—C9—N3—C14	−146.51 (19)
C16—C12—C13—C9	82.2 (2)	C11—C10—N3—C9	−26.8 (3)
C11—C12—C13—N6	127.84 (19)	N4—C10—N3—C9	156.08 (19)
C16—C12—C13—N6	−106.2 (2)	C11—C10—N3—C14	142.9 (2)
C11—C12—C16—C21	−164.9 (2)	N4—C10—N3—C14	−34.2 (3)
C13—C12—C16—C21	74.1 (3)	C9—C13—N6—O2	6.9 (3)
C11—C12—C16—C17	15.9 (3)	C12—C13—N6—O2	−164.43 (18)
C13—C12—C16—C17	−105.0 (2)	C9—C13—N6—O1	−174.0 (2)
C21—C16—C17—C18	−1.0 (4)	C12—C13—N6—O1	14.6 (3)

### Hydrogen-bond geometry (Å, º)

**Table:**

*D*—H···*A*	*D*—H	H···*A*	*D*···*A*	*D*—H···*A*
O3—H3*A*···N1^i^	0.82	1.97	2.785 (13)	179
N4—H4*B*···O3	0.86	2.26	2.902 (11)	132
C6—H6*A*···O2	0.97	2.13	2.783 (3)	124
C7—H7*B*···O2^ii^	0.97	2.57	3.378 (3)	141

Symmetry codes: (i) *x*, −*y*+1/2, *z*−1/2; (ii) −*x*+1/2, *y*−1/2, *z*.
